# Nontyphoidal Salmonella Septic arthritis of the elbow in a healthy infant

**DOI:** 10.11604/pamj.2015.22.357.7680

**Published:** 2015-12-11

**Authors:** Abdullah Saleh Al Nafeesah

**Affiliations:** 1 Pediatric Infectious Diseases Fellow, King Abdulaziz Medical City Riyadh, Saudi Arabia

**Keywords:** Nontyphoidal salmonella, septic arthritis, healthy, infant

## Abstract

A case of rarely encountered nontyphoidal Salmonella septic arthritis of the elbow in an infant with no preexisting disease is reported. Salmonella etiology was not suspected in this case, and the diagnosis was made only after bacterial isolation. Aspiration of the infected joint with radiological guidance initially failed to give a good clinical response. Arthrotomy was done with intravenous cefotaxime for 4 weeks followed by 2 weeks oral ciprofloxacin therapy to which the child responded favorably. Up to our knowledge this is the first case of nontyphoidal salmonella elbow septic arthritis in an infant in Saudi Arabia to be reported in the English literature.

## Introduction

Nontyphoidal Salmonellae Infections are a major cause of diarrheal disease in children accounting for 20% of the two million deaths that occur annually due to diarrheal illnesses. Bacteremia and focal extraintestinal nontyphoidal salmonella infections do occur with variable rates. They are higher in persons at the extremes of age, the immunocompromised, and those with hemoglobinopathies [1–3]. The rate of septic arthritis due to *Salmonella* (typhi and non-typhi strains combined) is estimated to be 0.1% to 0.2% [4]. Treatment usually consists of surgical intervention plus antibiotics therapy.

## Patient and observation

Our patient is a previously healthy 11 month old Saudi female infant, who was brought to our emergency room with a one week history of fever and left elbow swelling for 3 days. There was no history of vomiting or diarrhea. No history of trauma. No history of contact with sick patient. Past medical history was unremarkable with no previous hospital admission. No family history of hemoglobinopathy or immunodeficiency. No history chronic medication use or immunosuppressive therapy. On physical examination she was febrile with a temperature of 39.6°C. Over all she was looking well with left elbow swelling, hotness, redness, tenderness and limitation of active and passive movement. No other joint involvement. Other systemic examinations were not contributory. Her initial complete blood count showed a WBC of 14.21 x 10^9^/L, hemoglobin 104 gm/L and platelet 1140 x 10^9^/L. Erythrocyte sedimentation rate was 130 mm/hr and C reactive protein 57 mg/L. Blood culture was negative. Left elbow X-Ray showed mild amount of joint effusion with no evidence of fracture or dislocation. Ultrasound of left elbow showed 2 x 3 centimeter moderate joint effusion with synovial thickening and nodularity. Joint aspiration was done by interventional radiologist then the patient was started on vancomycin. Magnetic resonant imaging of left elbow ruled out osteomyelitis. Aspirated synovial fluid analysis revealed a WBC of 1111 and RBC 55625. Synovial fluid culture grew nontyphoidal salmonella group D sensitive to ampicillin, ceftriaxone, ciprofloxacin and trimethoprim/ sulfamethoxazole. Therefore vancomycin was stopped and cefotaxime was started. Three stool cultures were negative for salmonella. Because the isolated organism was salmonella, a sickle cell screening was done and came to be negative. Evaluation by Immunology team was obtained and their impression were unlikely to be immunodeficient child. After starting cefotaxime, her fever subsided but swelling and limitation of movement persisted. Ultrasound repeated at 10 days of hospitalization and showed thick elbow joint collection with synovial thickening and increased vascularity suggestive of active inflammation. Left elbow arthrotomy was done, pus was drained, joint was irrigated and a drain was placed. Three days later, the drain was removed and physiotherapy was started. Following this intervention, she improved significantly with resolution of all signs of inflammation. Cefotaxime was stopped after 4 weeks and she was discharged in good condition on oral ciprofloxacin for an additional two weeks. Upon discharge erythrocyte sedimentation rate and C reactive protein were within normal range. She was seen in outpatient three months after discharge and was found to be well.

## Discussion

Although the main presentation of nontyphoidal Salmonella is gastroenteritis, extraintestinal invasive diseases are not uncommon especially in certain susceptible hosts like; infants aged less than 12 months, immunocompromised patients and those with hemoglobinopathy. Galanakis et al showed that invasive disease occur in 21.4% of immunocompromised chidren in comparison to 3.5% in immuocompetent ones [5]. The commonest extraintestinal presentation is bacteremia occurring in 5 to 10% of the affected patients and around 5% of those with bacteremia may have focal infection with a predilection for bones, meninges and lung. Septic arthritis of the elbow caused by nontyphoidal salmonella is rare in healthy children. In his report Galanakis et al found among 15 immunocompetent children who had invasive nontyphoidal salmonellosis only one had septic arthritis. To the best of our knowledge, nontyphoidal salmonella septic arthritis has not previously been reported in the English literature from Saudi Arabia or Gulf countries. In our case, this diagnosis was unexpected as she had no antecedent gastroenteritis and she was apparently having no chronic underlying disease. In most reported cases, Salmonella septic arthritis is either associated with immunodeficiency, hemoglbinopathies or related to area where salmonella infection is endemic [6]. Our patient had no significant medical illness since birth, nor was he immunocompromised. We also did not identify any concurrent infections at other sites that might have attributed to the invasive infection. Salmonella species enter the bloodstream readily, and blood cultures should be considered whenever Salmonella infections are suspected or diagnosed. Blood culture may be positive in 10%-20% of cases only. It was negative in our patient. Gram-negative bacillary arthritis is known for its poorer prognosis compared to gram-positive joint infections [7]. In addition to antibiotic therapy, needle aspiration and/or arthrotomy and drainage are always required. Our patient did not show significant improvement until arthrotomy and drainage were done. The duration of therapy is usually prolonged ranging from 4-6 weeks with intravenous therapy given until the clinical signs resolve followed by oral therapy to complete the duration of therapy.

## Conclusion

Elbow septic arthritis caused by nontyphoidal Salmonella is very rare among healthy children. Therapy requires both surgical drainage and administration of appropriate antibiotics. Response to therapy might be prolonged compared to other causative organisms.

## References

[1] Zaidi E, Bachur R, Harper M (1999). Non-typhi Salmonella bacteremia in children. Pediatr Infect Dis J..

[2] Torrey S, Fleisher G, Jaffe D (1986). Incidence of Salmonella bacteremia in infants with Salmonella gastroenteritis. J Pediatr..

[3] Zarkowsky HS, Gallagher D, Gill FM, Wang WC, Falletta JM, Lande WM (1986). Bacteremia in sickle hemoglobinopathies. J Pediatr..

[4] Lee SC, Yang PH, Shieh WB, Lasserre R (1994). Bacteremia due to non-typhi Salmonella: analysis of 64 cases and review. Clin Infect Dis..

[5] Emmanouil Galanakis, Maria Bitsori, Sofia Maraki, Christina Giannakopoulou, George Samonis, Yiannis Tselentis (2007). Invasive non-typhoidal salmonellosis in immunocompetent infants and children. International Journal of Infectious Diseases.

[6] Shimoni Z, Pitlik S, Leibovici L, Samra Z, Konigsberger H, Drucker M (1999). Nontyphoid Salmonella bacteremia: age-related differences in clinical presentation, bacteriology and outcome. Clin Infect Dis..

[7] Pegues DA, Miller S, Mandell GE, Douglas RG, Bennett JE (2010). Principles and Practice of Infectious Diseases. Seventh.

